# RNA-based therapies: A cog in the wheel of lung cancer defense

**DOI:** 10.1186/s12943-021-01338-2

**Published:** 2021-03-19

**Authors:** Parvez Khan, Jawed Akhtar Siddiqui, Imayavaramban Lakshmanan, Apar Kishor Ganti, Ravi Salgia, Maneesh Jain, Surinder Kumar Batra, Mohd Wasim Nasser

**Affiliations:** 1 Department of Biochemistry and Molecular Biology, University of Nebraska Medical Center, Omaha, NE-68198 USA; 2 Fred & Pamela Buffett Cancer Center, University of Nebraska Medical Center, Omaha, NE-68198 USA; 3 Division of Oncology-Hematology, Department of Internal Medicine, VA-Nebraska Western Iowa Health Care System, Omaha, NE 68105 USA; 4 Division of Oncology-Hematology, Department of Internal Medicine, University of Nebraska Medical Center, Omaha, NE 68198 USA; 5 Department of Medical Oncology and Therapeutics Research, City of Hope Comprehensive Cancer Center and Beckman Research Institute, Duarte, CA 91010 USA; 6 Eppley Institute for Research in Cancer and Allied Diseases, University of Nebraska Medical Center, Omaha, NE-68198 USA

**Keywords:** Lung cancer, RNA interference, Antisense oligonucleotides, anti-miRs, mRNA-vaccine

## Abstract

Lung cancer (LC) is a heterogeneous disease consisting mainly of two subtypes, non-small cell lung cancer (NSCLC) and small cell lung cancer (SCLC), and remains the leading cause of death worldwide. Despite recent advances in therapies, the overall 5-year survival rate of LC remains less than 20%. The efficacy of current therapeutic approaches is compromised by inherent or acquired drug-resistance and severe off-target effects. Therefore, the identification and development of innovative and effective therapeutic approaches are critically desired for LC. The development of RNA-mediated gene inhibition technologies was a turning point in the field of RNA biology. The critical regulatory role of different RNAs in multiple cancer pathways makes them a rich source of targets and innovative tools for developing anticancer therapies. The identification of antisense sequences, short interfering RNAs (siRNAs), microRNAs (miRNAs or miRs), anti-miRs, and mRNA-based platforms holds great promise in preclinical and early clinical evaluation against LC. In the last decade, RNA-based therapies have substantially expanded and tested in clinical trials for multiple malignancies, including LC. This article describes the current understanding of various aspects of RNA-based therapeutics, including modern platforms, modifications, and combinations with chemo-/immunotherapies that have translational potential for LC therapies.

## Introduction

Lung cancer (LC) remains one of the primary causes of cancer-related death in men and women globally [1]. In 2020, approximately 228,820 new cases and 135,720 deaths due to LC had been reported in the United States alone [1]. LC is categorized into non-small cell lung cancer (NSCLC) and small cell lung cancer (SCLC). These two main subtypes have prominent intra-tumor heterogeneity and are further classified based on mutations and drivers [2, 3]. The majority of LC (~80-85%) fall in the category of NSCLC that includes adenocarcinoma, squamous cell carcinoma, and large cell carcinoma [4, 5]. Nearly 10-15% of cases belong to SCLC, categorizing into SCLC-A, SCLC-N, SCLC-Y, and SCLC-P subtypes [6, 7]. The statistics of the last two decades showed that the 5-year survival for NSCLC remains less than 20%, and for SCLC, it is nearly 5% [1, 6, 8, 9]. Some of the routinely investigated oncogenes for targeting in NSCLC include Kirsten rat sarcoma viral oncogene homolog (KRAS), epidermal growth factor receptor (EGFR), and echinoderm microtubule-associated protein-like 4-anaplastic lymphoma kinase (EML4-ALK). Genes implicated in SCLC include poly [ADP-ribose] polymerase (PARP), delta-like protein 3 (DLL3), aurora kinases, and vascular endothelial growth factor (VEGF) [10–13]. Approximately 30% of LC patients harbor activating KRAS mutations, making it a potential drug target for LC therapy. However, mutant KRAS targeting drugs have been under development for many years and are only now being evaluated in clinical trials [8, 14]. Similarly, treatment with tyrosine kinase inhibitors in the patients harboring EGFR mutations has been relatively ineffective in improving the overall survival (OS) [12, 15–18]. Similar gaps exist in SCLC therapies: for example, most patients develop resistance against chemotherapies, and due to the restricted expression of receptor antigens (PD1/PD-L1), immunotherapies show a narrow range of activity [19–23].

The major reason for the failure of currently available therapeutic approaches is the development of drug resistance associated with gene mutations, cancer stem cells, overexpression of oncogenes, and deletion or inactivation of tumor suppressor genes [10, 19, 24–28]. The collective outcomes suggested that the ‘tried-and-true’ therapeutic regimen to save LC patients is lacking and remains anticipated. To overcome these limitations, there is a rapidly growing interest in the field of RNA interference (RNAi) and RNA-based therapeutics, as several studies have shown that silencing of specific genes or overexpression of therapeutic proteins can serve as an effective combination modality with chemo- or immunotherapy [29–37]. The last decade witnessed the utilization of RNA therapeutics with chemotherapy and immunotherapy and emerges as an active research hotspot for the development of different types of cancer therapies [38]. The combination of adoptive cell transfer (ACT) therapy with self-delivering RNA interference (RNAi) was developed to down-regulate the expression of checkpoint proteins by degrading the respective mRNAs before their translation to proteins [39, 40]. These combinations also overcome drug resistance and improve the efficacy of chemo-/immunotherapy [39, 41]. RNA therapeutics can modulate multiple pathways, including gene silencing and overexpression, manipulation of enzyme kinetics, sensitization, and immune activation [37, 42, 43].

Additionally, advances in the field of noncoding RNAs have established their role in normal cell physiology or regulation of different molecular pathways, and studies demonstrating the direct role of noncoding RNAs in various pathologies have promoted the development of RNA-based therapeutics [44–48]. The RNA therapy-related studies suggested that these molecules have an immense potential to regulate multiple cellular pathways by inhibiting various genes [43, 49]. The ease of simultaneous targeting of multiple pathways provides an edge to the RNA-based therapeutics platform to target the different aspects of cancer such as tumor growth, metastasis, and drug resistance [47, 48, 50–53].

The current cancer treatment modalities, including surgery and chemotherapy, are far from ideal approaches, especially for the advanced stage tumors, as most of the tumors exhibit mutational diversity [7, 54–56]. These mutational heterogeneities play a significant role in cancer progression, chemoresistance, and immune escape [54, 55, 57]. Thus, instead of conventional targeted therapies (that include protein as a drug target), RNA-based treatment strategies are potentially superior, as they have a diverse target range with enhanced drug-like properties for cancer therapies [38, 58]. Several approaches have been employed to modulate gene-function at RNA level in the cancer cells, including base editing, small molecules targeting RNA, employment of synthetic antisense oligonucleotides (ASOs), and exogenously expressed mRNAs [42, 43, 59]. The promise of RNA-based therapeutic modalities is underscored by the successful outcomes of mRNA vaccine approach in treating the disease caused by the SARS-CoV-2 virus (COVID-19), and US Food & Drug Administration (FDA) approval of Patisiran (first RNAi-based treatment for hereditary transthyretin amyloidosis) and Givosiran (RNAi-drug for acute intermittent porphyria), and provide a strong rationale to explore RNA moieties as a novel therapeutic strategy for cancer [37, 60–62]. The recent advancements in terms of time, safety, pharmacokinetics, and potency further provide support for exploring RNA toolbox to develop potential anticancer therapies. This review article surveys the classification, applications, and recent progress of RNA-based treatments, including combination with first-line chemotherapy and immunotherapy for LC field advancing RNAs as therapeutic agents, the available preclinical and clinical studies with the future sequel to reach the patients.

### Platforms for RNA based cancer therapeutics

RNA-based therapeutic strategies have emerged as an alternative to the conventional protein-based therapies that are difficult to pursue, as adapter proteins or transcription factors. These types of protein molecules can be regulated by modulating mRNA levels or translation of proteins [53, 63]. The primary focus of oligonucleotide-based therapeutics includes gene silencing or activation and splice modulation that provides an extended range of potential targets beyond the conventionally accessible pharmacological strategies. These modalities follow the universal Watson–Crick base pairing rule of complementarity, thus providing the direct interrogation of different putative target sequences. Therefore, it is easy to rationalize, design, and screen-specific leads if the primary sequence of the target gene is available. To achieve the desirable functions (such as gene silencing, splice modulation, transcript degradation, translational activation, or antigen synthesis), different oligonucleotide-based platforms, including antisense oligonucleotides, RNA-interference molecules, and mRNA transcripts, have been developed. We discuss each platform in detail in the following sections.

### Antisense oligonucleotides

**Fig. 1:**
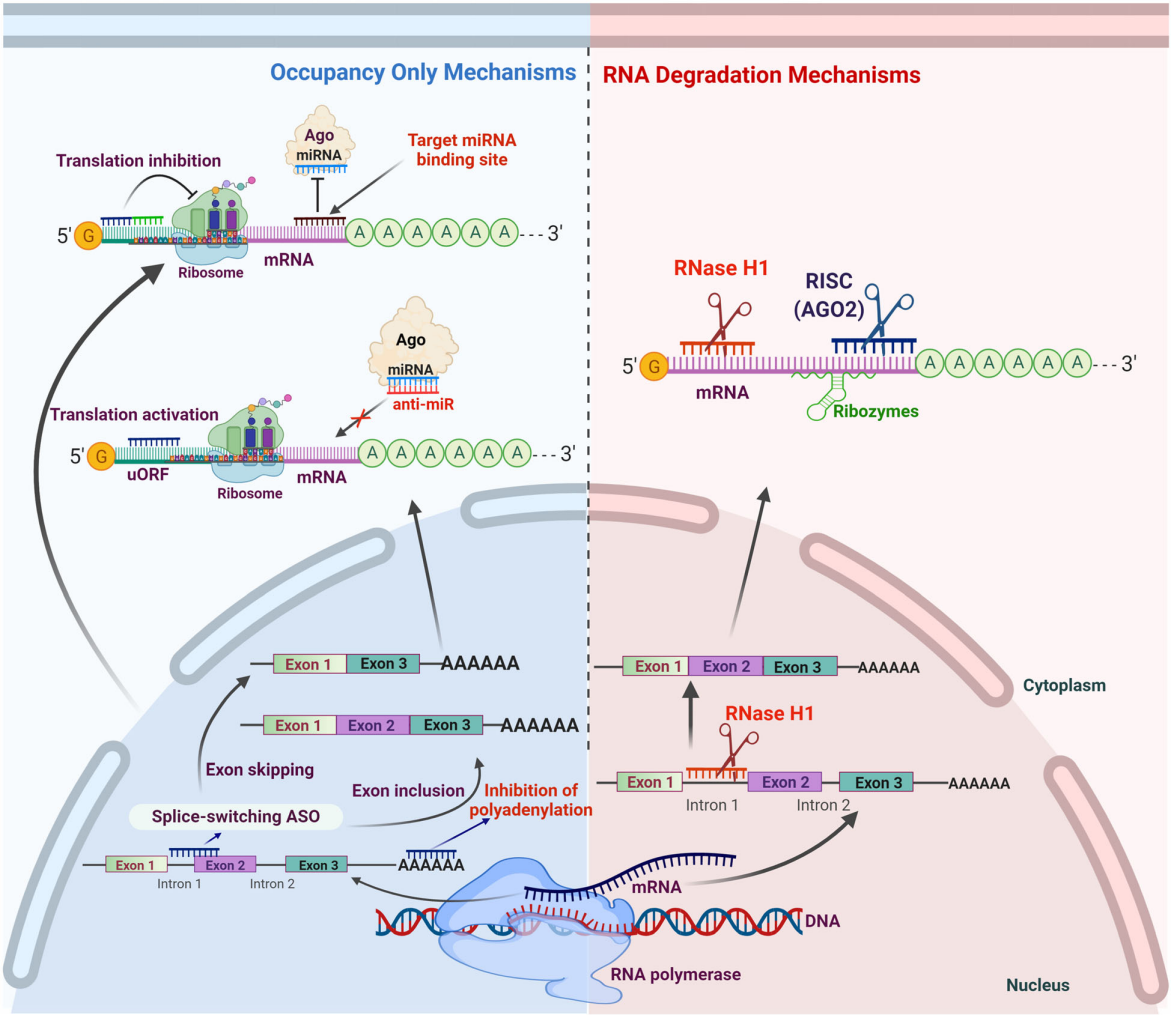
Different mechanisms of action for antisense oligonucleotide mediated gene silencing. Based on post-hybridization events, antisense oligonucleotides can modulate the expression of target gene through two different mechanisms 1) Occupancy-only mechanisms 2) RNA degradation mechanisms. In occupancy-only mechanisms, ASOs binding with target RNAs does not result in RNA degradation. It modulates the gene expression in several ways: splicing modulation using splice switch ASOs to perform exon-skipping or exon inclusion; inhibition of mRNA polyadenylation; translational modulation through non-DNA-like ASOs that base pair with mRNA, either to inhibit translation, for example, steric blocks or to activate translation by binding to inhibitory elements like upstream open reading frames (uORF). For the inhibition of miRNA-related function, these ASOs can also modulate miRNA either by base pairing with miRNA (anti-miRs) or by occupying miRNA-responsive elements (MRE) on target mRNA to nullify the effect of a particular miRNA. On the other hand, the ASOs in RNA degradation pathways trigger the target mRNA cleavage either by RNase H1 or siRNA-mediated AGO2 RISC complex and ribozymes mediated cleavage

Antisense oligonucleotides (ASOs) are ~18-30 nucleotide long, single-stranded, synthetic polymers of nucleic acids with diverse chemistries [44, 64]. The ASOs are small molecule drugs that target mRNAs based on complementary base pairing and interfere with different aspects of gene expression and regulation. These nucleotide sequences can interfere with DNA unwinding, transcription, mRNA splicing, gene expression/translational profile of target genes through different mechanisms [38, 58, 65]. Based on their mechanism of action the ASOs can be divided into two subcategories; one-acts by promoting RNA cleavage and degradation (either by ribonuclease H1 (RNase H1) or argonaute 2), and the other is occupancy-only mediated regulation, sometimes referred to as steric block (Fig. 1) [58, 64].

Binding of ASOs to the target RNA cleaves the target at ASO binding site, facilitating the degradation of target RNA and thus downregulating gene expression (Fig. 1). This is one of the most widely used approaches for downregulation of genes where overexpression is associated with the manifestation or progression of disease [58, 66]. RNase H1 is a highly selective endonuclease that specifically acts on the RNA of the RNA-DNA heteroduplex [58, 67, 68]. The detailed enzymatic and cellular functions of RNase H1 have been uncovered now, and the substrate specificity of RNase H1 is continuously utilized for the development of RNA-based therapeutics [43, 44, 68, 69]. In mammalian cells, the distribution of RNase H1 is ubiquitous and found in the cytoplasm, mitochondria, and nucleus [69, 70]. It serves various genomic functions, including DNA repair, resolution of R-loops, removal of pre-mRNAs associated with chromatin, transcriptional termination, maintenance of genome integrity, and removal of Okazaki fragment-associated RNA [44, 69–74]. Interestingly, the ASOs designed to utilize endonuclease activity as the mechanism of their action must possess a stretch of at least five DNA-nucleotides. Thus, currently used ASOs that follow RNase H-competent mechanisms are based on the patterns of DNA ‘gapmer’, a hybrid type of oligonucleotide sequence where the central stretch of DNA known as ‘gap’ is inserted between chemically modified RNA flanking sequences (that helps in target binding), Fig. 1. The main advantage of using RNase H1 based ASOs is that it makes the targeting of nuclear transcripts (for example, pre-mRNAs and long non-coding RNAs) easy. These are less accessible to other approaches like small interfering RNA (siRNA) [58].

**Fig. 2:**
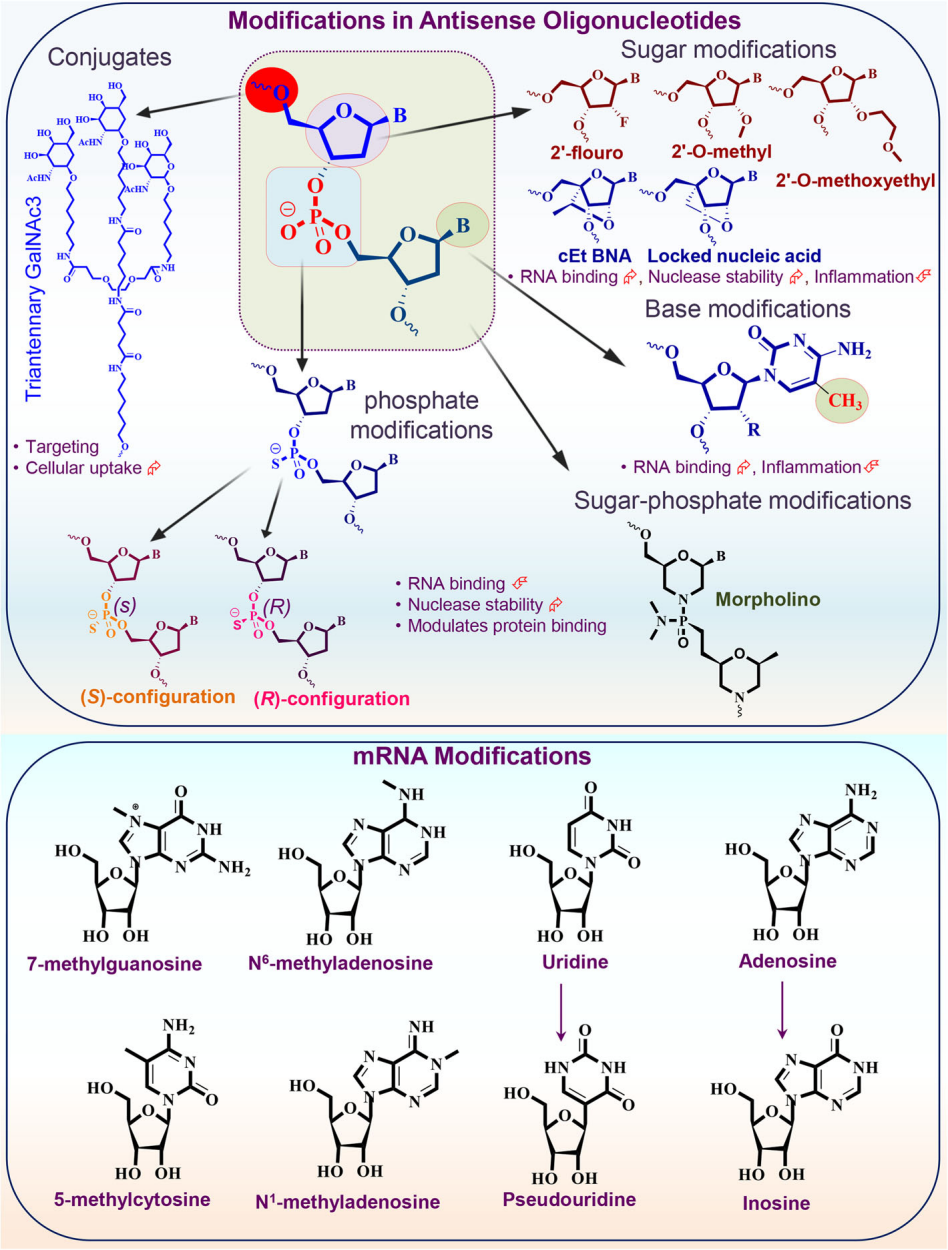
Common chemical modifications in antisense oligonucleotides and RNA oligonucleotides. Different types of RNA modifications or RNA analogs have been identified and evaluated in antisense mechanisms. A representative structure of dinucleotide is shown with marked positions where oligonucleotides are commonly modified. Commonly used modifications in ASOs consist of sugar modifications, base modifications, phosphate modifications, internucleoside linkage modifications, and conjugates of small or large molecules (as shown in the upper panel). Along with the structural modifications, the therapeutic properties of several key modifications are also highlighted. Specific internal RNA modifications are shown in the lower panel of figure (uridine-to-pseudo uridine and adenosine to-inosine transition are also presented). cEt BNA: (S)-constrained ethyl bicyclic nucleic acid

Steric block or occupancy-only mediated mechanism utilizes high-affinity ASOs to bind with the target RNA without inducing the direct degradation of target RNA [66, 75] (Fig. 1). This class of ASOs consists of nucleotides that do not form RNA-DNA duplex, which acts as a substrate for RNase H1 or Ago2. Therefore, to avoid the formation of RNase H substrates and unwanted cleavage of target RNA, the ASOs must be modified chemically or comprises of a mixture of different nucleotide chemistries, generally called ‘mixmers’ in such a way that stretches of consecutive DNA-like nucleotides can be avoided [64, 75]. Some common chemical modifications include thiophosphoroamidate, thiophosphoroamidate morpholinos, nucleoside moieties, and peptide nucleic acid attachments (Fig. 2) [76, 77]. Steric block ASOs bind with the specific sequence of target and work by modulating the translation, processing of RNA, splicing, RNA-protein interactions, and interactome of target RNA [65, 78, 79]. The most common application of these ASOs is to manage the selective exclusion or inclusion of exon(s) through the modulation of alternative splicing (for example, exon skipping and inclusion), Fig. 1 [80–82]. Interestingly, the ASOs steric block approach can be used for corrupting the target splice variant, where the exon skipping method hinders or downregulates the translation of the target transcript (Fig. 1) [83, 84]. The splice correction/inclusion approach has been used to correct or restore the translational frame to rescue the synthesis of therapeutic proteins [81, 85–89]. The ASOs perform this function by masking the splicing signals, making target invisible to the spliceosome, and finally alter the spliceosome’s splicing decisions [58, 81, 90].

Earlier, the antisense approaches were mainly used to downregulate the gene expression or translation, but not broadly as an alternative for the situations where the overexpression of beneficial proteins was required as a therapeutic strategy. More recently, the combinatorial investigation of several approaches like ASOs, micro-/si- RNAs to increase the target RNA expression or protein translation has revolutionized the RNA-based therapeutic strategies for different diseases, including cancer [44, 46, 65]. Further studies have established the utilization of ASOs for the targeting of microRNAs, and ultimately an efficient approach to enhance protein production (Fig. 1) [65, 79, 91, 92]. The microRNAs (short RNAs consisting of 21-23 nucleotides) inhibit the gene expression or protein translation and control associated gene networks. These observations have inspired the development of ASOs targeting microRNAs that block or repress their binding properties with target RNA transcripts, resulting in the translational escalation of microRNA-regulated genes (Fig. 1) [65, 79]. As microRNAs possess cell- or tissue-specific activities to inhibit the translation of multiple RNAs or targets, blocking of a single microRNA can alter the expression of different proteins. Alternatively, the utilization of specific ASOs against 5`-untranslated regions of mRNAs (that generally represses translation through upstream open reading frames or stem-loop structures) is a more precise and targeted approach to increase the protein production of associated RNA targets [44, 65, 79]. Alternative splicing is a beneficial strategy for generating protein diversity. ASOs can be utilized to induce isoform switching for promoting the expression of therapeutic/beneficial proteins and/or inhibiting the expression of disease-associated proteins (Fig. 1). Based on these mechanisms, three ASOs have received FDA approval for splice-switching: golodirsen, usinersen, and eteplirsen [58].

### RNA interference

The seminal paper published by Andrew Fire and Craig C. Mello in 1998 suggested the role of double-stranded RNAs in post-transcriptional gene silencing through a mechanism known as RNA interference (RNAi) and revolutionized the field of gene silencing [93]. This study contributed to the understanding of gene silencing and/or expression-related puzzles in fungi and plants and hit out the field by establishing the central role of non-coding RNAs in gene expression. Later, two independent studies from Elbashir et al., [94] and Caplen et al., [95] reported that small size double-stranded RNAs (approximately 19-22 nucleotides in length) having sequence homology to the silenced gene are the key mediators of sequence-specific post-transcriptional gene silencing in animals and plants. These small/short interfering RNAs (siRNAs) are processed from longer dsRNAs by ribonuclease III and maintain a characteristic structure (with 5'-phosphate/3'-hydroxyl ends) with a 2-nucleotide 3'-overhang on each duplex strand [94, 95]. These studies established that the siRNA molecule induces RNAi in mammalian cells without undesired interferon responses and is now accepted as a simplistic universal biological tool for gene silencing studies. RNAi is a mechanism used by the cells to downregulate gene expression in genetic abnormalities and infections [38]. Hence, RNAi approaches were explored and adapted as a potential therapeutic strategy for treating different diseases, including cancer [38, 47, 96–98].

**Fig. 3:**
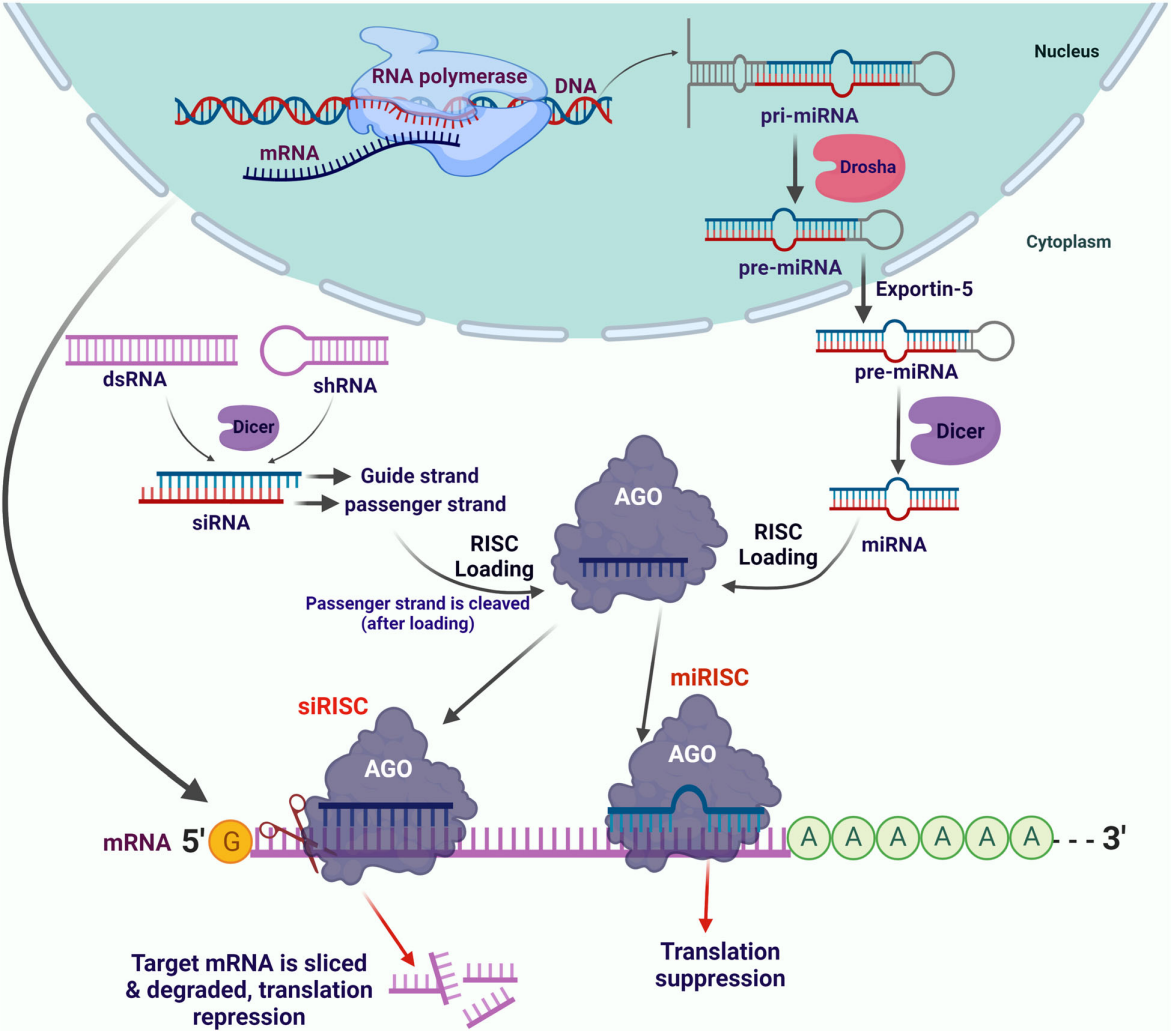
Schematic illustrations for siRNA/miRNA biogenesis and, the mRNA inhibition via siRNA and miRNA-mediated mechanisms

The potency, flexibility, and diversity of the RNAi or siRNA approach are alluring for prospective drug development targeting proteins that remain undruggable through classical approaches of small molecule inhibitors [99–101]. RNAi-mediated therapeutic approaches silence genes by utilizing the natural machinery of the targeted cells. These approaches include siRNAs, small hairpin RNAs (shRNAs), microRNAs (miRNAs), long double-stranded RNAs processed through Dicer, and small specific sequences synthesized to meet the RNAi criterion. Double-stranded siRNAs (ds siRNA) are pro-drugs-like molecules consisting of the complementary duplex of sense and antisense strand. Interestingly, the sense strand (passenger strand) of siRNA formally satisfies the definition of drug delivery vehicle; it is non-covalently associated with antisense or the guide strand that is complementary to the target RNA/transcript, protects it from degradation and helps in the loading of antisense strand to Ago2 (Fig. 3) [101, 102]. After loading to Ago2, the sense strand is removed before performing the pharmacological activity. The antisense strand guides the Ago2-mediated RNA-induced silencing complex (RISC) to the target site, and the complete complementarity of siRNA with the target leads to the cleavage (known as slicer activity) and silences gene expression (Fig. 3) [102–105]. Ago2 is an RNA endonuclease with RNase H domains, but unlike RNase H1, it cleaves RNA in RNA-RNA duplex (not DNA-RNA duplex) [106]. Ago2 complex plays an important role in facilitating the binding of antisense strand to the target transcript, and thus, Ago2 has become a key regulator for efficient RNA-based pharmacological mechanisms with different features [102, 107]. The loading of siRNA or specifically antisense strand into Ago2 is a very efficient process, but for effective binding and cleavage activities, Ago2 has some strict structural requirements [108]. For example, availability of 5'-phosphate or phosphate analog and comparatively fewer modifications were allowed at 2' site, located at the distal site of seed sequence (nucleotide sequence that recognizes RNA targeting site) [64, 109].

Ago2 also prolongs the duration of action as once the antisense strand is loaded, it retains the strand for a more extended period [107, 110]. The localization of Ago2 is cytoplasmic; hence, siRNAs are used as prominent tools for targeting cytoplasmic RNAs (Fig. 3**)** [64, 102]. Studies suggest that some modifications in conventional siRNA sequences or designs can improve the pharmacological benefits like enhanced potency and reduced off-targeting. Few examples of atypical siRNAs include single-stranded RNAs [111, 112], divalent siRNAs [113], self-delivering siRNAs [114], small internally segmented siRNAs [115], and Dicer substrate siRNAs [116]. The outcomes of RNAi studies recently led to the FDA approval of two siRNA based therapeutics named Patisiran and Givosiran [61, 62].

### MicroRNAs

MicroRNAs (miRNAs or miRs) are short non-coding RNAs that trigger endogenous RNAi by regulating the stability or inducing mRNA degradation. The miRNAs have diverse role in various physiological and pathophysiological progressions, including cell-cycle progression [117], cancer development and progression [118–120], metabolism [121], diabetes [122], infectious diseases [123, 124], muscular dystrophy [125], and immunity [126]. Therefore, miRNAs are an important class of putative drug targets. The biogenesis of miRNAs follows a systematic process; the initial or primary miRNA strand is transcribed in the nucleus (Fig. 3) [127, 128]. The miRNA hairpin structure, embedded within the primary miRNA strand, is sequentially processed by DROSHA and DICER (both belong to the RNase III family) and finally emerges as a mature miRNA consisting of 21-22 nucleotides [127, 128]. Mature miRNA sequence is then loaded to the RISC complex and modulates gene expression by binding with the 3'-untranslated region (UTR) of the target gene (Fig. 3). The inhibition of gene expression is directly dependent on miRNA’s complementarity to that of target mRNA [38]. In addition to the inhibition of gene expression, miRNAs also modulate transcriptional regulation. Recent studies showed that miRNAs regulate the methylation of CpG islands in the promoter region of different genes and, thus, directly regulate the transcriptional regulations through epigenetic modifications [129–132]. The primary mode of action for miRNA and siRNA is similar, as both form RISC complex for targeted gene silencing (Fig. 3). The main difference is that siRNAs degraded or inhibited mRNA translation with 100% complementarity and thus precisely follow target specificity. In contrast, miRNAs usually bind with incomplete complementarity and perform gene silencing through slicer-independent pathways. The miRNAs target 3'-UTR of mRNA and suppress the gene expression or decrease its stability. Because miRNAs can act through low complementarity; thus, they could have multiple targets, but the primary safety check is the restriction of imperfect base pairing; otherwise, one miRNA can affect thousands of genes.

Interestingly, the ASOs have also been developed and employed for miRNA inhibition through direct binding to the small RNA molecules in the RISC complex, and these ASOs are known as antagomirs or anti-miRs (Fig. 1) [133–135]. Miravirsen (also known as SPC3649) was the first anti-miRNA drug designed to treat chronic hepatitis C virus (HCV), and it targets the activity of liver-specific miR-122 [136]. The 5'-UTR of HCV RNA consists of two binding sites for miR-122, which stabilizes the viral RNA [137, 138]. Miravirsen inhibits this binding by sequestering miR-122, making it readily available for exonuclease degradation, decreases replication, and thus reduces the viral load [139, 140]. However, viral recovery in patient serum and resistance to Miravirsen was observed, along with the development of new mutations [140, 141]. Similarly, another anti-miR drug, RG-101, was designed (by Regulus Therapeutics) against miR-122 and used to control HCV infections but failed to improve overall outcomes in clinical trials [142]. Similarly, RG-101 induced viral rebound, along with the substitutions in the binding regions of miR-122 (in the 5' UTR of the HCV genome) and developed resistance [142]. Outcomes of another clinical trial suggested that treatment with RG-101 restores the natural killer (NK) cell population that controls HCV infection [143]. A recent clinical trial suggested the potential of a combination regimen of RG-101 and GSK2878175 (a non-nucleoside NS5B polymerase inhibitor) to develop a single-visit cure for HCV patients [144].

Several groups are also developing anti-miR drugs against miR-21, miR-17, miR-155, and miR-29 for cancer, kidney, and other diseases [58, 145–148]. These miRNAs (especially miR-21) have a diverse role in lung cancer establishment, progression, and metastasis, so these anti-miRs can also be utilized as an effective therapy for lung cancer. Steric block ASOs are also being developed to target specific miRNAs. These oligonucleotides obstruct the regulatory interactions of miRNAs with target mRNA (Fig. 1), thus providing an important strategy to downregulate the activity of diseases specific miRNAs [149]. The details of related reports and implications of anti-miRs/ASOs in lung cancer therapies are discussed in forthcoming sections.

### mRNA platforms

The fundamental step of the central dogma of molecular biology is that mRNA carries the information from DNA and transfer to the protein synthesis factories [150]. Protein molecules are the ‘workhorses’ of the body as nearly every function (normal and disease-related) of the human body is performed by different proteins [151]. Interestingly, the mRNA is equally critical as DNA because the human body would never utilize the genetic code if mRNA is not available, or proteins will never be synthesized. Besides, normal physiological functioning of the human body, downregulation of therapeutic proteins or upregulation of diseases associated proteins, and entry of foreign proteins lead to the disease condition [151, 152]. The important functional role associated with different proteins ultimately clues towards the development of protein-targeted drugs or therapies.

Due to several difficulties in protein targeting, researchers moved to DNA-based gene therapy. However, the stumpy likelihood of genome integration and transient nature makes it challenging to use in the clinics [153–155]. On the other hand, mRNA is a molecule that overcomes these two major pitfalls (targeting and genome integration) and has emerged as a strong alternative to conventional gene therapy strategies [153, 155–157]. Additionally, it uses natural or homegrown cell machinery for protein synthesis that return properly folded mature therapeutic protein with all post-translational modifications, thus providing better opportunities over recombinant proteins [157]. The treatment strategies based on mRNA therapies involve the implications of specific mRNA sequences into the patient’s body and utilization of cellular machinery to synthesize specific proteins involved in the disease progression. This method is applicable in multiple conditions, as it can be used to overexpress specific proteins whose downregulation is associated with disease and could be used to elicit an antigenic response by inducing the expression of specific antigens [37, 51, 158].

However, initial studies suggested that *in-vitro* transcribed/synthesized mRNA molecules are less stable as they are readily accessible to nucleases and easily detected by toll-like receptors (TLRs) and activate dendritic cells (DCs) that generate innate immune response [159–161]. To understand the immunogenic responses associated with synthetic mRNAs, researchers have incorporated several modifications in RNA nucleosides [162–164]. A very interesting study by Kariko et al. showed that modification of mammalian RNA nucleosides (for example, 5-methylcytidine, N6-methyladenosine, 5-methyluracil, pseudouridine, and N7-methylguanosine) decreases the immunomodulatory signals and DCs exposed to these modified mRNAs reduced activation and cytokine production compared to DCs exposed to unmodified RNAs [165]. This is also a defense mechanism utilized by innate immune response of the human body to bacterial or other foreign non-mammalian RNAs. These organisms have less abundance of modified nucleosides that potentially activate TLRs expressing cells and DCs [165]. Thus, modifications in nucleosides overcome RNA-mediated activation of DCs. This approach can potentially affect the design and development of mRNA-based therapies.

Following their synthesis, RNA molecules can undergo more than 150 different chemical modifications in the cells that impact their stability, distribution, and other post-transcriptional events; collectively, these modifications are known as ‘epitranscriptome’ or RNA epigenetic modifications [166–169]. Some of the common modified mRNA nucleosides are N6-methyladenosine (m6A), 5-methylcytidine (m5C), N7-methylguanosine (m7G), pseudouridine (s2U), inosine, and many 2′-O-methylated nucleosides (a part of the 5′-terminal cap), as shown in Fig. 2. Each chemical modification plays a specific role like, m6A enhances mRNA turnover, regulates embryonic stem cell development, favors RNA decay, pre-mRNA splicing, adipogenesis, and prostate cancer bone metastasis [170–174]. Similarly, s2U regulates the structure of RNA, increases stability, and alters translational efficiency [175, 176], while m5C induces codon rewiring and, in combination with other modifications, guides miRNA targeting [166, 177]. For translation, ribosomes scan mRNA transcripts at the 5′ UTR within Kozak sequences to identify start codon, but the length of 5′ UTR, presence of cis-elements, and m6A modulate ribosome scanning and finally regulate the translational efficiency [172, 178, 179]. Interestingly, some transcripts that retain m6A in their 5' UTR can be translated in a 5′ cap-independent manner due to the direct binding of 5' UTR m6A with the eukaryotic initiation factor 3 (eIF3), which alone is enough to recruit the 43S ribosomal complex and initiate translation [180]. Inhibition of N6-methylation in adenosine specifically decreases the translation of mRNA transcripts consisting 5'UTR m6A. The cap-independent translation mechanism was studied for heat shock protein 70 (Hsp70) mRNA, and it was observed that cellular stress induces a global rearrangement of m6A in the transcriptome Hsp70, making more mRNAs with m6A in the 5' UTR [180, 181]. Thus, m6A in 5' UTR helps translate the mRNA under stress conditions through bypassing the dependency of 5' cap-binding proteins and suggests that such RNA modifications can be incorporated while designing and optimizing therapeutic RNAs/mRNAs.

**Fig. 4:**
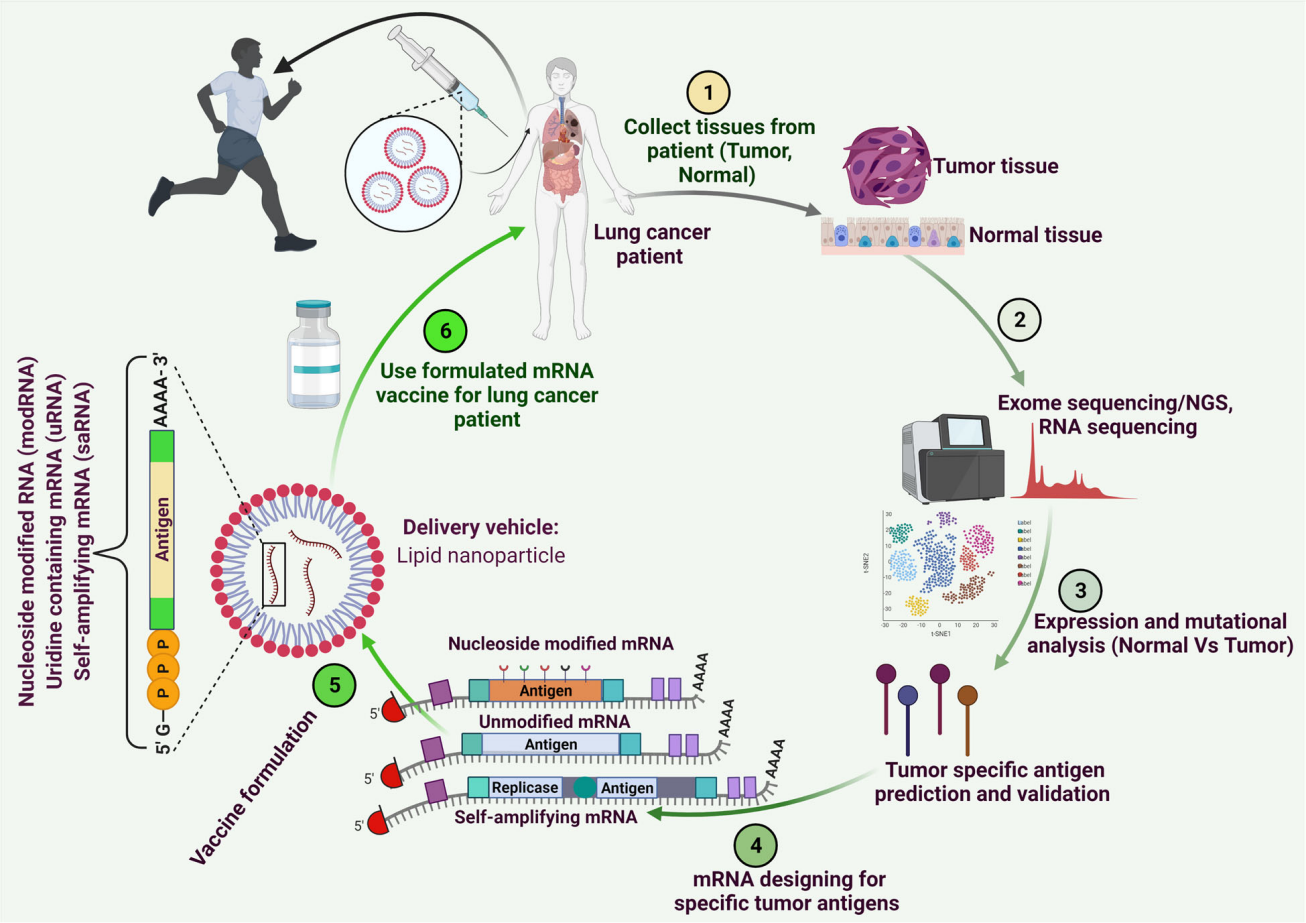
Schematic illustrations for mRNA-based LC vaccine development. The aim of mRNA vaccine development was started with a comparative analysis of exome of LC tissue and normal tissue to identify potential tumor-specific antigen(s). The detailed analysis coupled with high throughput methods enables the verification of identified antigen(s)/neoantigen(s) specific to LC. The mRNA sequence(s) specific to tumor antigens will then be synthesized, modified, and cloned into appropriate plasmids for the mRNA transcription. The liposome formulations (or other appropriate vehicles) of the final, optimized mRNA(s) encoding LC-specific antigens will be injected into LC patients to elicit a prominent anticancer immune response for the destruction of LC tumors

With the diverse applications of RNA-based therapies and the potential to translate into clinics, synthetic mRNAs have emerged as a powerful tool and alternative to conventional therapies/vaccines. In recent years, significant advancement has been achieved to develop mRNA-based therapeutics for immune-oncology, protein replacement therapies, and vaccine development [37, 60, 182]. Indeed, mRNA-based vaccine formulations developed by Pfizer and Moderna were developed and approved in record time to combat coronavirus disease 2019 (COVID-19) caused by the global outbreak of severe acute respiratory syndrome coronavirus 2 (SARS-CoV-2) [182–184]. The successful safety and efficacy outcomes of these vaccines are likely to enhance the enthusiasm and trust, and likely to dictate the future course of RNA-based therapeutics in general. Based on recent mRNA-based cancer vaccine studies in melanoma [51] and other infections like COVID-19 mRNA-based vaccine, the developmental route map for the mRNA-based LC vaccines is outlined in Fig. 4.

### Implications of RNA-based platforms in LC

#### ASOs in LC therapy

The ASOs have broad applications for different anticancer therapies, including LC. The implications of anti-angiogenic therapies for lung squamous cell carcinoma are limited and associated with adverse side effects [185–187]. Recent evidences established the role of long noncoding RNAs in tumor progression and VEGF modulation that guide the potential of RNA-based therapies for the development of anti-angiogenic strategies for LC [188–195]. Overexpression of LINC00173.v1 was associated with proliferation, tumorigenesis, migration of vascular endothelial cells, and poor overall survival in squamous cell carcinoma patients [196]. LINC000173.v1 upregulates the expression of VEGFA through miR-511-5p sponging and inhibition of LINC000173.v1 using ASOs resulted in decreased tumor growth and enhanced cisplatin sensitivity [196].

A recent study demonstrated the potential of ASOs to downregulate the expression of metadherin, which plays a role in T cell exhaustion and WNT signaling [197]. Downregulation of metadherin in cell lines and spontaneous mouse models using locked nucleic acid (LNA) modified ASOs significantly decreased LC progression and metastasis [197]. Similarly, miR-21 is among the top differentially upregulated microRNAs in NSCLC and regulates cell growth, proliferation, migration, invasion, apoptosis, and drug resistance [198–202]. Ge et al. recently reported the use of phosphorothioate ASOs to inhibit miR-21 expression and found that these ASOs decrease the proliferation of NSCLC cells and induce apoptosis through caspase-8 pathway activation [198].

KRAS is a frequently mutated gene in different cancers, and activating mutations in KRAS are observed in 20% of human tumors, including NSCLC [54, 203–206]. KRAS is a highly desirable target for cancer therapy; however, the development of pharmacological small molecules for mutant KRAS remains challenging, and most of the identified inhibitors are still in early-stage clinical trials [14, 205–207]. AZD4785 is a high-affinity KRAS mRNA-targeting therapeutic ASO that selectively decreases mutant KRAS mRNA as well as protein. Unlike the inhibitors of RAS-MAPK pathways, this depletion did not activate the feedback loop of the MAPK pathway. AZD4785 downregulated the effector pathways and selectively decreased the proliferation of cells harboring mutant KRAS [208]. The systemic injection of AZD4785 to NSCLC mice xenografts and patient-derived xenografts harboring mutant KRAS inhibited KRAS expression and induced strong antitumor activity. This suggested that AZD4785 and other novel ASOs are innovative therapeutic approaches for treating KRAS- and other mutated oncogenes.

Wang et al. reported the construction of novel polyethylene glycol (PEG) ligated antisense therapeutic oligonucleotides that form a bottlebrush-like structure consisting of PEG side chains, DNA backbone, and overhangs of antisense oligonucleotides [209]. These formulations possess PEG at high density on the surface, which decreases the undesired interactions of ASOs with DNA and proteins (protecting from enzymatic degradation) and enhances the chances of antisense overhangs to hybridize with target mRNA, thereby reduce protein expression. This study evaluated KRAS targeting by these PEGylated ASOs in lung cancer. These modified ASOs had higher inhibition efficacies than conventional hairpins, and the antisense molecules decreased the proliferation of LC cell line expressing mutant KRAS (G12C) gene [209]. These ASOs showed enhanced *in vivo* retention time (due to high biological compatibility of PEG) and represent an important strategy to improve biopharmaceutical efficacies and translational applicability of ASOs mediated therapies.

ADP-ribosylation factor like 4C (ARL4C), a member of small GTP-binding protein family, is frequently overexpressed in adenomatous hyperplasic lesions (precursors to adenocarcinoma) and lung cancer [210]. ARL4C promotes cell proliferation and is a potential therapeutic target for lung cancer [211–213]. ARL4C expression is directly correlated with different histologic stages (adenocarcinoma, minimally invasive adenocarcinoma, and invasive adenocarcinoma) and is associated with a poor prognosis. ASOs targeting ARL4C (ASO-1316) showed decreased RAS-related substrate activity, inhibited cell proliferation and migration, and suppressed nuclear import of Yes-associated protein-1 (YAP-1) in lung cancer cell lines bearing KRAS and EGFR mutations. In addition, ASO-1316 reduced tumorigenicity of KRAS/EGFR mutated lung cancer cell lines in orthotopic mice models [210]. This study established the role of ARL4C in the initiation of premalignant lesions, tumor progression and development, and demonstrated the utility of ASO-1316 as a potential therapeutic agent for LC patients with ARL4C overexpression, regardless of mutation status.

A recent study demonstrated that ASOs attached with deoxyadenosine (dA_40_) can form complex with β-glucan schizophyllan (SPG) [214], and ASOs-dA_40_/SPG complex can be recognized by Dectin-1 (a β-glucan receptor) expressed on lung cancer cells and antigen-presenting cells (APCs) [215, 216]. ASOs-dA_40_/SPG targeting KRAS inhibited KRAS expression in Dectin-1 expressing LC cells and correspondingly decreased cell growth. This ASOs-dA40/SPG complex enhanced the cytotoxic effect of gemcitabine due to the ability of dA_40_ moiety to directly interact with gemcitabine. Interestingly, after internalization, this interaction dissociates, and gemcitabine is easily released from the complex [214]. It means that conjugation of SPG and dA40 bearing ASOs can also serve as potential carriers for gemcitabine and other structurally similar drugs. Due to this dual ability of ASOs-dA40/SPG complexes to target KRAS and enhance gemcitabine efficacy, it will be of interest to evaluate these formulations in clinical trials.

Signal transducer and activator of transcription 3 (STAT3) is a central molecule for oncogenic/non-oncogenic signaling. STAT3 overexpression is associated with the progression of various cancers, including LC, making it a potential target for cancer therapy [217]. AZD9150, an advanced ASO consisting of ethyl-modifications, was designed to target STAT3 as it is challenging to target transcription factors through small molecule inhibitors [218]. Preclinical evaluations demonstrated that AZD9150 decreased the expression of STAT3 and exhibited prominent antitumor effects in several preclinical cancer models of lymphoma and LC. In a phase I clinical trial (NCT01563302) for diffuse large B-cell lymphoma (DL-BCL) AZD9150 was well tolerated and exhibited efficacy in a subset of heavily pre-treated patients [219]. Currently, combination studies of AZD9150 with immune checkpoint inhibitors are in progress. Therapeutic silencing of STAT3 can also be achieved by targeting STAT3 binding to target sites. CS3D is a cyclic 15-mer oligonucleotides decoy corresponding to the response element of STAT3 target genes [220]. The CS3D decoy was tested on EGFR inhibitor-resistant NSCLC cells and resulted in the downregulation of STAT3 targeted c-MYC gene at mRNA and protein levels. Further, CS3D inhibited cell proliferation, colonization and, increases apoptosis *in vitro,* and reduced tumor growth along with c-MYC expression *in vivo* [220]. Thus, targeting of STAT3 with RNA-based oligonucleotides is a promising alternative for small molecules chemical inhibitors to develop effective therapies for LC.

High expression of Bcl2 and Akt1 promotes the growth, proliferation, and apoptotic evasion in LC, and Bcl2 is a potential therapeutic target for both NSCLC and SCLC [221–223]. G3139 and RX-0201 are ASOs that target Bcl2 and Akt-1, respectively, but have exhibited limited efficacy in clinical trials due to inadequate delivery. These ASOs were modified using the ‘Gapmer’ strategy and 2'-O-methyl modifications at 5' and 3' ends. To enhance the targeting and delivery, cancer cell-specific lipid nanoparticles (conjugated with transferrin receptor-targeting T7 peptide) were synthesized with these modified ASOs [224]. The ASO co-loaded nanoparticles exhibited enhanced colloidal stability, high encapsulation with smaller particle size, and higher cellular uptake. The T7-ASO-lipid nanoparticles decreased the expression of Bcl2 and Akt-1in LC cell lines, exhibited superior antitumor effects, and improved the overall survival (OS) in LC xenograft bearing mice [224]. The *in vitro* activity of gapmer based G3139 ASO lipid nanoparticles for Bcl2 was also evaluated [225]. The G3139-GAP (with 2'-O-methyl nucleotides) was incorporated into DOTAP/egg PC/cholesterol/Tween 80 lipid nanoparticles, and anticancer efficacy was studied in A549 cells and xenograft mouse models. These gapmer based ASOs lipid nanoparticles reduced the Bcl2 expression at mRNA as well as protein level in cell lines and tumors, and inhibited tumor growth, and improved OS [225].

Self-renewing tumor-initiating cells (TICs) or cancer stem cells mainly contribute towards tumor initiation, recurrence, and treatment resistance [226–228]. The overexpression and activity of glycine decarboxylase (GLDC) maintain TICs and is possibly responsible for tumorigenesis of NSCLC [229]. GLDC is a key member of the glycine and serine metabolic pathway, regulating pyrimidine metabolism and cancer cell proliferation. No therapeutic molecule is available for GLDC; hence ASOs could be a novel strategy to downregulate GLDC [230]. The splice-modulating steric-block ASOs were designed to induce exon-skipping to disrupt the open reading frame of GLDC encoding transcripts and induce nonsense-mediated degradation. These GLDC steric blocks inhibited cell proliferation and colonization of LC cell lines, and NSCLC TICs derived tumor-spheres. The candidate GLDC ASOs decreased the tumor growth of TICs derived xenografts in mice [230]. Overall, these reports suggested that ASOs hold strong promise to design and develop RNA-based therapeutic regimens for LC.

#### RNAi therapies in LC

RNAi moieties (siRNAs and miRNAs) effectively silence target genes by inducing mRNA degradation or inhibiting the binding sites of translational machinery (Figs. 1 & 3) [8, 38]. Cyclooxygenase-2 (COX-2) is an important drug target in LC that regulates cancer progression, metastasis, metabolism, and tumor immunity [231–234]. However, the available COX-2 inhibitors have failed to show clinical efficacy. Instead of direct COX-2 inhibition, knockdown of delta-5-desaturase (D5D) offers a unique approach that limits the formation of arachidonic acid (a substrate for COX-2) and promotes the peroxidation of dihomo-γ-linolenic acid leading the production of 8-hydroxyoctanoic acid (8-HOA) [235]. Pang et al. incorporated the siRNA of D5D with epithelial cell adhesion molecule (EpCAM) aptamers into three-way junction RNA nanoparticles that exhibited target specific accumulation, D5D knockdown, and formation of 8-HOA in lung cancer cell lines and mouse models [236]. These D5D siRNA-loaded nanoparticles inhibited the proliferation of lung cancer cells and induce apoptosis by suppressing YAP1/TAZ axis.

Single nucleotide polymorphism (SNPs) and lncRNAs play a prominent role in LC [237–239]. Analysis of LC risk-associated SNPs and lncRNAs, identified an oxidative stress-responsive serine-rich 1 antisense RNA1 (OSER-AS1) as a prognostic biomarker and therapeutic target [240]. Downregulation of OSER1-AS1 in tumor tissues was associated with poor OS in NSCLC patients. Myc represses the promoter of OSER-AS1, which is also targeted by RNA binding protein ELAVL (embryonic lethal, abnormal vision, Drosophila)-like 1 (ELAVL1) and hsa-miR-17-5p at the 3'-end. OSER1-AS1 acted as a decoy for ELAVL1 and inhibited its interaction with target mRNA. Treatment with OSER1-AS1 resulted in the inhibition of growth and metastasis of xenograft LC tumors.

Two independent studies performed on lncRNA nicotinamide nucleotide transhydrogenase-antisense RNA1 (NNT-AS1) showed that overexpression of NNT-AS1 is correlated with poor prognosis of NSCLC [241, 242]. NNT-AS1 upregulation decreases miR-22 by sponging and is associated with increased expression of FOXM1 and YAP-1. Knockdown of NNT-AS1 attenuates cell proliferation, invasion, migration, induces apoptosis, and suppresses *in vivo* tumor growth. Further, NNT-AS1 contributes to drug resistance in NSCLC through MAPK-slug signaling [243]. Thus, NNT-AS1 is a potential RNA-based therapeutic target and prognostic marker for NSCLC. Wanjun et al., reported the analysis of non-canonical small non-coding RNAs (sncRNAs) using peripheral blood mononuclear cells of human and identify a unique ‘disease RNA code’ named TRY-RNA signature composed of distinct tRNA-derived small RNAs (tsRNAs), rRNA-derived small RNAs (rsRNAs), and YRNA-derived small RNAs (ysRNAs) [244]. This TRY-RNA signature helps to differentiate between LC and pulmonary tuberculosis, and thus possess diagnostic implications for LC screening [244]. A recent study demonstrated the potential of PD-L1 siRNA encapsulated gold nanoparticles for the imaging and treatment of LC [245]. These nanoparticles downregulate the expression of PD-L1 in NSCLC cell lines and xenograft studies and serve as photothermal agents for LC photothermal therapy [245]. Thus, it demonstrates the theranostic application of siRNAs in LC when combined with suitable photothermal agent. KDM3A is lysine-specific demethylase that increases the expression of DCLK1 by reducing the methylation of H3K9me2. It was recently demonstrated that bone marrow mesenchymal stem cell-derived extracellular vesicles (BMSC-EV) encapsulated let-7i miRNA decreases LC growth by suppressing the KDM3A-DCLK1-FXYD3 axis [246]. This study established KDM3A is a direct target of let-7i, and the high expression of KDM3A and DCLK1 is associated with reduced expression of let-7i. *In vivo* BMSC-EV derived let-7i downregulated the KDM3A, and decreased tumor growth [246].

In addition, extracellular miRNAs also serve as diagnostic biomarkers for LC [247]. A study involving NSCLC patients, patients with benign nodules, and healthy controls demonstrated the utility of miR-520c-3p and miR-1274b in the identification of NSCLC risk factors [248]. The panel of these two-miRNA has the potential to differentiate between NSCLC and benign nodules and suggested the importance of extracellular miRNAs for diagnostic utilization in NSCLC [248]. Similarly, another recent study demonstrated the clinical significance of circulating/serum exosomal miR-let-7e as a biomarker for NSCLC metastasis [249]. Analysis of serum exosomes and tumor tissues from NSCLC patients demonstrated that miR-let-7e was low, and suppressor of variegation 3-9 homolog 2 (SUV39H2) was high in NSCLC tissues and was associated with low OS. The ectopic overexpression of miR-let-7e or treatment with serum-derived exosomes (miR-let-7e is high in serum-derived exosomes) decreased cell viability, migration, invasion, and delayed *in vivo* tumor growth by targeting the SUV39H2-LSD1-CDH1 axis [249].

The therapeutic utility of miRNAs is also being investigated for SCLC. For example, low expression of miRNA-195 has been observed in SCLC [250]. Low miRNA-195 and high Rap2C were associated with low OS in SCLC patients. Overexpression of miRNA-195 decreased the proliferation of SCLC cells through Bax upregulation and Bcl2 downregulation. Further, this study identified the binding site for miRNA-195 in the Rap2C mRNA. Overexpression of miRNA-195 in SCLC cell lines inhibited the activation of the MAPK pathway by decreasing the expression of Rap2C and inducing apoptosis [250].

#### Combination of small RNAs with chemotherapy in LC

Small RNA therapies substantially affect the growth of LC, and thus targeted RNA combination therapies may be used to improve therapeutic response. Several efforts have been made to improve the utilization and formulations of RNA-based cancer therapeutics, such as the use of nanoparticles as a delivery vehicle [158, 251]. Interestingly, the nanoparticles-based delivery systems not only protect the small molecule RNAs from degradation but also facilitate the evaluation of different combinational approaches to develop effective LC therapies. Combination of chemotherapy with anti-angiogenesis therapies is an attractive approach against NSCLC [252, 253]. Integration of these two different targeting strategies (anti-angiogenesis and chemotherapy) is a promising approach to simultaneously target the tumor vasculature and tumor cells. Two independent studies reported the utility of combining VEGF siRNA with two different chemotherapeutic drugs [252, 253]. Zhang et al reported the efficacy of coupling VEGF siRNA with gemcitabine using lipid-calcium-phosphate nanoparticles that possess cell-specific targeting [252]. Compared to gemcitabine or VEGF siRNA alone, systemic administration of co-targeting nanoparticles resulted in improved response in subcutaneous as well as orthotopic mouse models of NSCLC. The combination of VEGF siRNA and a gemcitabine-induced significant decrease in tumor growth and tumor microvessel density with minimal *in-vivo* toxicity [252]. Similarly, novel nanoparticles containing tripeptide lipids, sucrose laurate, folate-PEG_2000_-DSPE were used to encapsulate paclitaxel and VEGF siRNA [253]. These nanoparticles showed substantial specificity and anti-tumor activities in cell lines and mouse models of LC. In addition, these formulations show improved bioavailability and led to a decrease in the effective therapy dose, thereby reducing toxicity [253].

KRAS is the most common oncogenic mutation in patients with NSCLC and is associated with recurrence and poor survival [3, 254]. KRAS mutations are leading to the constitutive activation of the KRAS, which suggested the potential of mutant K-*ras* inhibition, may for NSCLC treatment [255]. The approach combining siRNA and miRNA provides innovative therapeutic opportunities to combat oncogenic KRAS and other oncogenic mutations in LC simultaneously. The lipid-based polymeric nanoparticle containing the siRNA for knocking-down oncogenic KRAS and overexpressing miR-34a (p53-regulated tumor suppressor miRNA) has shown promising therapeutic effects in lung cancer [33]. Additionally, this dual NP (miR-34a/siKras) in combination with cisplatin exhibited greater efficacy as compared to cisplatin alone, suggesting that miR-34a/siKras small RNA therapy can be combined with conventional chemotherapeutic approaches to improve LC therapy [33]. Furthermore, anti-mutant KRAS siRNA-loaded hybrid nanoparticles (AKSLHNs) have been shown to target the KRAS and inhibit the metastasis in a mouse model of lung cancer [256].

Similarly, most of the anti-EGFR therapies aim to target the EGFR mutations, and the status of the EGFR mutation determines the fate of such treatments [17, 257, 258]. In combination with TK inhibitors, siEGFR induced apoptosis and reduced NSCLC cell growth [259]. Recently, silencing of EGFR-TKs by a siRNA pool and simultaneously delivering paclitaxel by using tumor-targeted nanostructured lipid carriers resulted in enhanced tissue distribution and anticancer effects compared with monotherapy or non-targeted therapy [260]. Studies have indicated that miRNAs contribute to the resistance of TK inhibitor in EGFR mutated NSCLC tumors. Expression of miR-146b-5p was higher in the pleural discharge of treatment naïve patients as compared to EGFR TK inhibitor-resistant patients. Overexpression of miR-146-5p in the resistant cells enhanced their sensitivity to EGFR TK inhibitors. Similar observations were noticed in osimertinib resistant primary cancer cells in both EGFR-dependent and independent manner [261]. Mechanistically, miR-146b-5p targets interleukin 1 receptor-associated kinase 1 (IRAK1) by downregulating NF-κB and related cytokine production (IL-6 and IL-8). Thus, miR-146b-5p has the potential to target IRAK1/NF-κB signaling, regulating EGFR TK inhibitor resistance, and may help combat resistance associated with TK inhibitors [261]. MicroRNA-506 (miR-506) functions as a tumor suppressor in multiple cancers, including LC [262–264]. Haque et al. reported downregulation of miR-506-3p and Sonic Hedgehog (SHH) signaling pathway in erlotinib resistant NSCLC cell lines [265]. Overexpression of miR-506-3p inhibited SHH signaling pathway and modulated epithelial to mesenchymal transition [265]. This study identified the SHH pathway as a novel therapeutic target of miR-506-3p in EGFR TK inhibitor-resistant EGFR mutated LC cell lines.

A decreased expression of miR-3180-3p was noticed in NSCLC cell lines and tumor tissues [266]. Exosome-mediated delivery of miR-3180-3p decreased the proliferation and metastasis of NSCLC cells through forkhead box P4 (FOXP4). FOXP4 is an important target for EGFR mutated LC and is involved in the regulation of pulmonary gene expression. This suggests a possibility of a miRNA-mediated targeting approach for FOXP4 associated pathways as a potential treatment option in LC [267, 268]. miR-139-5p was shown to induce cisplatin sensitization of NSCLC cells [269]. The expression of miR-139-5p was downregulated in NSCLC compared to adjacent normal tissue, and reduced expression was associated with cisplatin resistance. Overexpression of miR-139-5p resulted in enhanced sensitivity to cisplatin, inhibited cell proliferation, and induced apoptosis by modulating the Homeobox protein Hox B2 (HOXB2) and PI3K-AKT-caspase-3 axis [269].

As discussed in ASOs section, VEGF is a key mediator of angiogenesis in most human tumors and is associated with tumor relapse, metastasis, and poor prognosis of NSCLC [270, 271]. Several targeted therapies for VEGF and VEGF receptor (VEGFR), including antibodies and small molecule inhibitors, have been evaluated in NSCLC. Bevacizumab is a recombinant humanized monoclonal antibody against VEGF-A that has been approved as first-line therapy for unresectable, recurrent, locally advanced, or metastatic NSCLC [272]. Although combining the bevacizumab with chemotherapy improved the overall survival in NSCLC, VEGF inhibitors have a short half-life and numerous side effects [273–276]. Recently, targeting of tumor angiogenesis via siVEGF was shown to be an effective strategy in metastatic NSCLC [277]. Further, co-administration of siVEGF and etoposide using cationic liposomes inhibited tumor growth and metastasis more effectively than monotherapy [278]. Further, ASO against LINC00173.v1 reduced the growth of lung squamous cell carcinoma and enhanced the sensitivity to cisplatin *in vivo* via modulating the VEGFA expression (for details, see ASO section) [196]. Ribonucleotide reductase large subunit (RRM1) is a key enzyme that plays a part in DNA synthesis, as it is required for deoxyribonucleotide synthesis, and overexpression of RRM1 is associated with LC [279, 280]. Adenoviral vector-based short hairpin siRNA targeting the RRM1 gene (Ad-shRRM1)-mediated inhibition of RRM1 augmented the sensitivity to gemcitabine, and combination treatment with Ad-shRRM1 and GEM exerted significantly better inhibitory effects in LC compared to monotherapy [281].

Bcl-2 is a well-known oncogene, is highly expressed in the majority of SCLC, and contributes to chemotherapeutic resistance [282]. G3139, a Bcl-2 ASO, along with paclitaxel was well-tolerated in chemo-refractory SCLC patients [283]. Further, G3139, in combination with carboplatin and etoposide showed promising results in SCLC patients [284]. However, a randomized phase II Study for Bcl-2 ASOs (Oblimersen) in combination with carboplatin and etoposide did not improve the clinical outcome in advanced-stage SCLC patients [285]. Another oligonucleotide against telomerase (Imetelstat) failed to improve the progression-free survival in advanced NSCLC patients [286]. Clusterin (Apolipoprotein J) encodes a chaperone protein that is highly overexpressed in NSCLC patients [287]. A Phase I study of custirsen (OGX-011), a second-generation ASO to clusterin combined with cisplatin and gemcitabine, showed a promising response in patients with stage IIB/IV NSCLC [288].

AXL is overexpressed in tumor tissue of NSCLC patients and associated with poor survival [289]. Growth arrest-specific protein 6 (GAS6) is a high-affinity ligand of AXL. Gas6/AXL signaling pathway is associated with tumor growth, metastasis, invasion, angiogenesis, drug resistance, and immune regulation, making AXL a potential target for several cancer therapies, including NSCLC [290–293]. Recently a combination of an EGFR targeting antibody (Cetuximab)-functionalized gelatin nanoparticle (GAb) and covalently conjugated AXL siRNA containing nanoconstruct (GAbsiAXL) showed higher potential for intercellular internalization, improved the siRNA stability, and increased the expression of tumor suppressor P53 in drug-resistant NSCLC cells [294]. Further, the combination of EGFR inhibitor (erlotinib) and GAbsiAXL synergistically enhanced apoptosis and inhibited cancer cell migration, demonstrating that RNA-based inhibition of AXL in the combination of chemotherapies may be beneficial in NSCLC [294].

Survivin is overexpressed in many cancers and regulates the several pathways required for cancer stem cells and tumor maintenance [295]. Thus, it is an exceptionally attractive target for cancer therapeutics [295]. A lipid-modified platinum-derivative-based nanoparticle delivery system for survivin siRNA in combination with cisplatin exhibited improved therapeutic efficacy in chemo-resistant LC model, suggesting that RNA therapeutics against survivin is a promising approach [296]. The polyglutamate-derived brush polymer-based silencing of survivin using si-RNA (PPGS/si-survivin polyplex) combined with cisplatin exhibited synergistic cytotoxic effects on drug-resistant LC cells [297]. Further, a pH based polyglutamate brush polymer (DMA-mPEG-b-PG-g-spermine, DPPGS) containing the dual siRNAs against MDR1 (siMDR1) and survivin (si-Survivin) enhanced sensitivity to the cisplatin. This combination appears to be a promising approach for overcoming multi-drug resistant (MDR) NSCLC [298]. Interestingly, survivin is also being investigated as a potential candidate for immunotherapy and vaccine development [299].

Aprinocarsen, a first-generation ASO, is a phosphorothioate oligonucleotide that targets human PKC-α mRNA and inhibits PKC-α expression [300–302]. In NSCLC, aprinocarsen has been extensively investigated as a single anticancer agent or in combination with various chemotherapeutic agents. In combination with chemotherapy, aprinocarsen showed promising activity in early phase studies, while higher toxicity was reported in phase III trials (Table 1**)** [300, 301]. Similarly, in SCLC, oblimersen failed to show benefit, either alone or in combination with chemotherapy (Table 1) [285]. The outcome of a phase II clinical trial, evaluating the efficacy of carboplatin and pemetrexed plus either apatorsen, a Hsp27 mRNA targeting ASO, or placebo showed no additional toxicity; however, no improvement was observed in treatment naïve patients with metastatic nonsquamous NSCLC [306].

**Table 1:** RNA based therapeutics in clinical trials in lung cancer

Drug combination	Affected Pathway	References
Custirsen + gemcitabine + cisplatin	Clusterin	**[**288**]**
Aprinocarsen + gemcitabine + cisplatin	PKC-a	**[**300**,** 303**,** 304**]**
Imetelstat + bevacizumab	Telomerase	**[**286**]**
LY2181308 + docetaxel	Survivin	**[**305**]**
Oblimersen + paclitaxel	Bcl2	**[**283**]**
Oblimersen + carboplatin + etoposide	Bcl2	**[**285**]**

Besides therapy and diagnosis, miRNAs also play a role in immunotherapy resistance [307]. Anti-PD1 immunotherapy, in combination with first-line chemotherapy, improves the overall outcomes of LC; however, long-term benefits of this regimen are frequently compromised due to resistance to anti-PD1 [308–310]. Guyon et al. recently developed a cellular model consisting of T-cell and cell lines of different cancers (glioblastoma, lung adenocarcinoma, breast cancer, and ovarian carcinoma) [307]. They used longitudinal blood samples from anti-PD1 treated patients and LC mouse model and demonstrated the enrichment of exosomal miRNA-4315 following anti-PD1 exposure to T-cells. The exposure of cancer cell lines to exosomal miRNA-4315 induced apoptosis resistance to chemotherapy via inhibition of Bim (a pro-apoptotic protein) expression. The introduction of ABT263 (a BH3 mimetic) bypassed this resistance. The analysis of patient blood samples suggested that miRNA-4315 and cytochrome-c levels help define the timeline to add ABT263 to enhance cell death and overcome anti-PD1 resistance [307]. This study established the role of exosomal miRNA-4315 for the stratification of LC patients developing anti-PD1 resistance and provide an alternative therapeutic option to utilize miRNAs for LC to modulate immunotherapy.

#### mRNA vaccines in LC

The primary objective of cancer vaccines is to elicit or boost cancer-specific immunity. Tumor antigens trigger cancer immune response, and identification and formulation of potential tumor antigens is a challenging task [51]. In the context of antigen formulation, mRNA-based approaches provide a promising way to design and synthesize antigens using intracellular machinery of the host/patient [37, 51]. DCs present tumor antigenic peptides to cognate T-cell receptors (TCRs) and induce tumor immunity and immunological memory [311]. The main targets of cancer vaccines include tumor-associated antigens (TAA) and cancer neoantigens. The atypically expressed proteins of tumors such as overexpression, different subcellular localization, tumor specific expression (which are normally sequestered in immune-privileged sites or express during certain differentiation stages) compared to normal tissues constitutes TAA. The recent success of immune checkpoint blockade and initial success of RNA-vaccine in Melanoma renewed the interest of researchers in RNA-based cancer vaccines [37, 51, 157, 312]. RNA-based vaccines have emerged as a promising substitute for conventional vaccines. The recent interim outcomes of a multicenter, open-label, dose-escalation phase 1 clinical trial (NCT02410733) of an intravenously administered liposomal RNA (RNA-LPX) vaccine initiated by Shahin et al., demonstrated the immunogenic potential of this mRNA-based vaccine in melanoma [51]. The cancer vaccine field is in the developing phase, and only a few studies are available, particularly for LC. A clinical trial involving patients with stage IV NSCLC showed the benefits of immunotherapy consisting of protamine-protected, sequence-optimized mRNA (BI1361849 or CV9202) encoding six NSCLC-associated antigens, including New York Esophageal Squamous Cell Carcinoma-1 (NY-ESO-1), MAGE-C1, MAGE-C2, survivin, 5T4, and Mucin-1), to induce targeted immune responses in combination with local radiation treatment [313]. The treatment was well-tolerated with minor side effects. BI1361849 increased antigen-specific immune responses in most patients, whereby antigen-specific antibody levels and functional T cells were increased by 80% and 40% of patients, respectively, supporting further clinical investigation [313]. Similarly, another phase I/IIa study also demonstrated that CV9201 was well-tolerated and enhanced immune response in stage IIIB/IV NSCLC patients [314]. These results suggest the importance of mRNA-based immunotherapy in combinations with immune checkpoint inhibitors in NSCLC. Similarly, an ongoing phase I/II study (NCT03164772) is evaluating the efficacy and safety of mRNA Vaccine (BI 1361849) in the combination of checkpoint inhibitors, anti-PD-L1 (durvalumab), and anti-CTLA-4 (tremelimumab) for the treatment of NSCLC.

## Conclusion and future perspective of RNA therapeutics

The utilization of RNA as a drug is a fundamentally novel approach to conventional small molecule inhibitors. The idea to translate RNA oligonucleotides' inhibition mechanism into clinics almost took four decades to become a reality. The recent FDA approvals of Givosiran, mRNA-1273-P301 (Moderna), and BNT162b1 (Pfizer-BioNTech) COVID-19 Vaccine [62, 315–317], have ushered the wave of RNAi or mRNA-based therapies into the mainstream of drug development. The outcomes of RNA-based treatments also open a novel direction of alternative therapeutic strategies to explore RNA moieties for cancer therapy development. The advances in the understanding of siRNAs/miRNAs are expected to facilitate the development of more effective ‘combinational approaches’ through multi-targeting properties of these small RNAs to treat cancer, as it is a multi-gene-associated problem. The potential of RNA therapies in precision genetics, like for the treatment of hereditary transthyretin amyloidosis [61] and acute intermittent porphyria [317], has raised enthusiasm for similar applications in cancer therapies.

The new generation ASOs with GalNAc conjugation (Givosiran) showed enhanced liver-specific delivery and more than twenty-fold enhanced potency for RNase H1 dependent ASOs [44, 317], suggesting a possibility to develop anticancer molecules to deal with organ-specific metastases of LC or other cancers. The success of targeted delivery and improved potency of ASOs provide a strong motive to identify ligands/conjugates that can enhance the potency and targeting in other tissues. Targeting is a major problem in cancer drug development, so such modifications also provide a window to further optimize the targeting of anticancer RNA drugs. However, RNA therapies can potentially reach the ‘golden-age’ in some diseases, but before we reach the goal, there are several challenges ahead, especially for cancer research. Some of the major pitfalls are targeted delivery, the stability of chemically synthesized RNAs compared to in-vitro/in-vivo transcribed RNAs, modulation of immune responses, and efficacy improvement. The field of RNA nucleoside modifications or epitranscriptomics is also under-investigated, including the identification of oligonucleotides that can target RNA modifications and the associated molecular pathways for the development of cancer therapies. Nevertheless, some of the ASOs or small RNAs did not proceed to the clinics, still putting forward the potential and implications of the strategy to further modify and optimize the RNA moieties for the development of effective therapies for LC. The outcomes of the studies also demonstrated the application and potential of combining chemotherapeutic drugs and RNAi tools or suggested the possibilities of coupling multiple antisense molecules into a single nanoformulation, aiming to expand the efficacies of LC therapies.

The recent outcomes of liposomal encapsulated mRNA-based tumor vaccines have been promising in melanoma (Lipo-MERIT trial, ClinicalTrials.gov identifier NCT02410733) [51]. This vaccine provides durable antigen-specific cytotoxic T-cell immune response alone or in checkpoint inhibitor (PD1) treated patients and provided vital evidence for the utilization of non-mutated commonly shared tumor antigens for the development of cancer vaccines. This initial success has raised hopes for cancer vaccine development, and the focus now is to identify tumor-associated antigens that can serve as potential antigenic targets for the tumors possessing high mutational burdens like NSCLC and SCLC.

## Data Availability

Not applicable, all information in this review can be found in the reference list.

## Abbreviations

NSCLC: Non-small cell lung cancer
SCLC: small cell lung cancer
RNAi: RNA interference
EGFR: Epidermal growth factor receptor
KRAS: Kirsten rat sarcoma viral oncogene homolog
TK: Tyrosine kinases
PARP: ADP-ribose polymerase
ACT: Adoptive cell transfer
DLL3: Delta-like protein 3
VEGF: Vascular endothelial growth factor
ASOs: Antisense oligonucleotides
RNase H1: Ribonuclease H1
siRNA: Small interfering RNA
IRAK1: Interleukin 1 receptor associated kinase 1
ARL4C: ADP-ribosylation factor like 4C
shRNAs: Small hairpin RNAs
miRNAs: MicroRNAs
RISC: RNA-induced silencing complex
m6A: N6-methyladenosine
m5C: 5-methylcytidine
m7G: N7-methylguanosine
s2U: Pseudouridine
KDM3A: Lysine demethylase 3A
DCLK1: Doublecortin-like kinase 1
FXYD3: FXYD domain-containing ion transport regulator 3
HOXB2: Homeobox protein Hox B2
SHH: Sonic Hedgehog
